# Residual Magnetic Field Testing System with Tunneling Magneto-Resistive Arrays for Crack Inspection in Ferromagnetic Pipes

**DOI:** 10.3390/s24113259

**Published:** 2024-05-21

**Authors:** Shuxiang Zhao, Junqi Gao, Jiamin Chen, Lindong Pan

**Affiliations:** 1 Qingdao Innovation and Development Base, Harbin Engineering University, Qingdao 266400, China; zhaoshuxiang@mgsdcyjy.cn; 2 Moganshan Institute of Geomagnetism Large Scale Scientific Facility, Zhejiang, Huzhou 313200, China; 3 State Key Laboratory of Transducer Technology, Aerospace Information Research Institute, Chinese Academy of Sciences, Beijing 100094, China; jmchen@mail.ie.ac.cn

**Keywords:** residual magnetic field, magnetization, crack detection, tunneling magneto-resistive (TMR), ferromagnetic pipes

## Abstract

Ferromagnetic pipes are widely used in the oil and gas industry. They are subject to cracks due to corrosion, pressure, and fatigue. It is significant to detect cracks for the safety of pipes. A residual magnetic field testing (RMFT) system is developed for crack detection in ferromagnetic pipes. Based on this background, a detection probe based on an array of tunneling magneto-resistive (TMR) sensors and permanent magnets is exploited. The probe is able to partially magnetize the pipe wall and collect magnetic signals simultaneously. First, a theoretical analysis of RMFT is presented. The physics principle of RMFT is introduced, and a finite element model is built. In the finite element simulations, the effects of the crack length and depth on the RMFT signal are analyzed, and the signal characteristics are selected to represent the crack size. Next, the validated experiments are conducted to demonstrate the feasibility of the proposed RMFT method in this paper.

## 1. Introduction

Steel pipes are widely used in oil and gas transportation due to their excellent performance, such as high strength, pressure resistance, and good impact toughness [1]. In steel pipes, carbon steel has ascendancy over stainless steel owing to low cost. However, carbon steel is a ferromagnetic material, which has relatively weak corrosion resistance compared with stainless steel [2]. Therefore, as a joint result of corrosion, pressure, and fatigue, carbon steel is more prone to cracks as time goes on. If these cracks cannot be found in time, many serious accidents, such as leakages, fires, and even explosions, will be caused by the propagation of these defects [3].

It is necessary to locate and evaluate the cracks in pipes for the safety of energy transportation, lives, and property. Previously, cracks in pipes have been successfully detected by some non-destructive testing (NDT) technologies. For instance, Piao et al. reconstructed the 3D profile of a defect in a Q235B steel pipe using magnetic flux leakage (MFL) signals [4]. However, the pipe wall is magnetized to saturation, which is the precondition in MFL testing [5]. As a consequence, the device for MFL is bulky and requires high-power consumption to produce a large enough magnetic field [6]. Wu et al. used ultrasonic waves to inspect the cracks with different inclined angles in a pipe [7]. Nevertheless, the coupling agent is indispensable in the ultrasonic testing (UT) method [8]. Eddy current testing (ECT) [9] and alternating current field measurement (ACFM) [10] are also applied to assess the defects in metal pipes. However, with respect to ferromagnetic pipes, the signals of deep defects are a weakness of the two technologies, due to skin effects [11]. In a word, there are inherent limitations to these mentioned technologies, which are used to detect defects in ferromagnetic pipes.

All aforementioned NDT methods acquire the characteristic signal of a defect by various sensors. In particular, coil is a frequently used scheme in electromagnetic NDT technologies, but it relies on the changing frequency of inducing a magnetic field to collect the signal [12]. So, the signal-to-noise ratio (SNR) in response to the signal is very low at low frequency [13]. Furthermore, winding the coil is complicated and onerous. In addition to coils, magnetic sensors based on the Hall effect [14], anisotropic magnetoresistance (AMR) [15], giant magnetoresistance (GMR) [16], and tunneling magnetoresistance (TMR) [17] are widespread in defect detection. Among these magnetic sensors, TMR has the largest magnetoresistance (MR) ratio, which can reach 472 and 804%, at room temperature and 5 K, respectively [18]. The sensitivity of TMR is at least 10 times that of AMR and GMR, let alone the Hall sensor, which processes the smallest sensitivity. The other key parameter of sensors is background noise, which determines the limits of detection. This indicator can achieve 150 $pT/\sqrt{Hz}$ at *f* = 1 Hz in TMR sensors [19]. Certainly, the linear range of TMR is narrower than coils and Hall sensors, which limits its application in strong magnetic fields.

Residual magnetic field testing (RMFT) represents an electromagnetic NDT technology capable of defect detection for ferromagnetic metals [20]. Similar to MFL, RMFT relies on a permanent magnet or exciting coil to magnetize a sample. However, the magnetized effects in the two methods are different. Namely, saturation magnetization is a precondition to using MFL technology, while RMFT requires only partial magnetization for the detected sample. In addition, some highly sensitive magnetic sensors, such as GMR and TMR, are hard to utilize in MFL [3]. The reason is that the saturated magnetic field is usually beyond the linear range of these sensors. Nevertheless, there are no limitations in RMFT, due to the weak magnetic field.

The contributions of this paper are as follows:

(1) An RMFT system is developed to detect the cracks in ferromagnetic pipes. An array of permanent magnets and TMR sensors are applied to build a detection probe, which can implement pipe for magnetization and signal acquisition simultaneously.
(2) An experiment is conducted using the designed RMFT system. The characteristics Δ*B_x_* and *l_x_* in RMFT signals can be used to express the crack size (length and depth), which validates the feasibility of the RMFT method.

The rest of the paper is organized as follows: First, in Section 2, the physical principles and a simulation model of RMFT for crack detection are introduced. Second, the RMFT system, the experimental results that verify the feasibility of the RMFT method, and the discussion are presented in Section 3. Finally, in Section 4, conclusions are drawn and future work is considered.

## 2. Theoretical Analysis

### 2.1. Physics Principle

**Figure 1:**
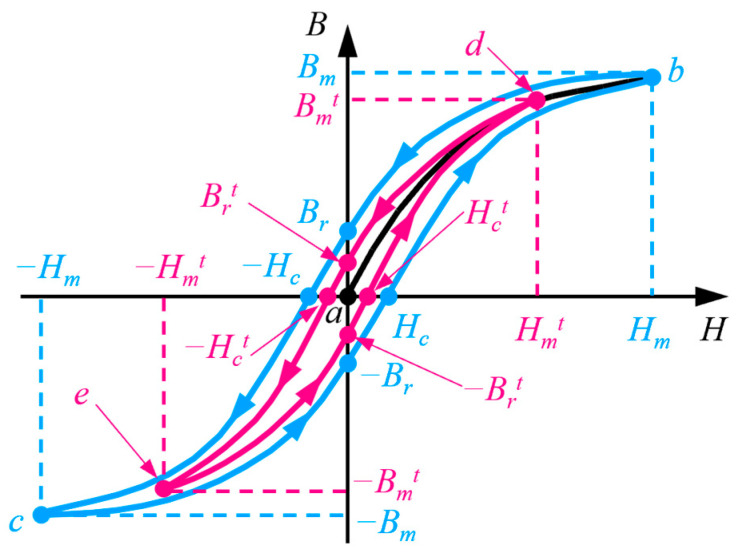
Magnetized curve of ferromagnetic material.

From the perspective of a magnetized curve, the saturated magnetization of ferromagnetic material can be divided into five distinct phases [21]. First, the external magnetic field *H* is steadily applied from 0 to the positive maximum value *H_m_*. Second, *H* is gradually decreased from *H_m_* to −*H_c_*, which is denoted as the negative coercive field strength. At this moment, the magnetic flux density in the ferromagnetic material is 0. Third, the *H* is sequentially reduced from −*H_c_* to the negative maximum value −*H_m_*. Fourth, the external magnetic field is reversely enhanced from −*H_m_* to *H_c_*. Finally, *H* is further strengthened and goes back to *H_m_*. The five processes form a closed curve (*a*–*b*–*c*–*b*), which is denoted as the saturated hysteresis curve, as shown in Figure 1. Correspondingly, the variation of the magnetic flux density in ferromagnetic material also has five stages. (1) The magnetic flux density *B* is increased with *H* from 0 to the positive saturation *B_m_*. (2) *B* is gradually attenuated to the residual flux density *B_r_*, and its direction is positive. (3) *B* is declined once more, and its value is changed from *B_r_* to the negative saturation −*B_m_*. (4) The magnetic flux density is augmented, and the value of *B* is varied from −*B_m_* to −*B_r_*. (5) *B* is ulteriorly increased and goes back to *B_m_*.

In terms of the unsaturated magnetization, the waveform of the hysteresis curve is similar with the saturated magnetization. However, the area of the hysteresis curve in unsaturated magnetization is clearly smaller than that in saturated magnetization. The rose-red line in Figure 1 denotes the hysteresis curve in unsaturated magnetization. The magnetized process is also accompanied by the reciprocating conversion of the external magnetic field (*H*). The arrival points of *H* are *a*, *d*, *e*, and *d*, successively. Correspondingly, the values of *H* are 0, $H_{m}^{t}$, −$H_{c}^{t}$,−$H_{m}^{t}$, $H_{c}^{t}$, $H_{m}^{t}$, respectively, where $H_{m}^{t}$ and $H_{c}^{t}$ denote the maximum magnetic field and the coercive field strength in the unsaturated hysteresis curve, respectively. Similarly, the magnetic flux density (*B*) is varied in order of the hysteresis curve (0–$B_{m}^{t}$–$B_{r}^{t}$–−$B_{m}^{t}$–−$B_{r}^{t}$–$B_{m}^{t}$). $B_{m}^{t}$ and $B_{r}^{t}$ refer to the maximum value and the residual value of magnetic flux density, respectively. 

**Figure 2:**
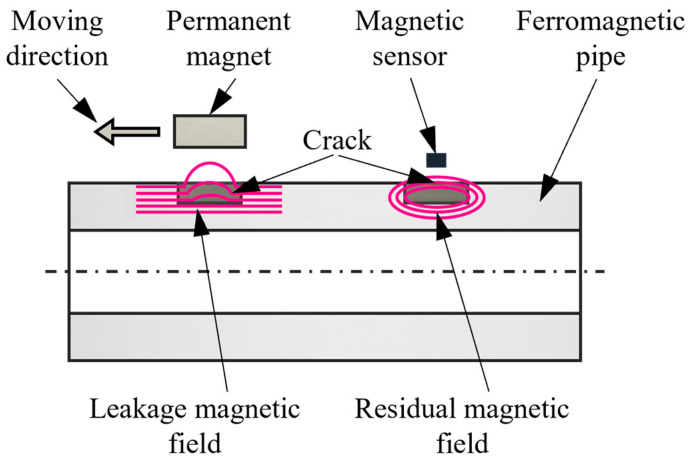
Principle of RMFT for crack detection.

The principle of RMFT for crack detection in ferromagnetic pipes is shown in Figure 2. The pipe wall is partially magnetized by a moving permanent magnet. Then, the magnet is removed, and the residual magnetic density is retained. As for the pipe wall, there is an in-and-out motion of the permanent magnet, namely, the increasing and decreasing process of the external magnetic field. Therefore, the residual magnetic flux density $B_{r}^{t}$ (or −$B_{r}^{t}$) can be stored in the pipe wall. When it is in the presence of a crack, the magnetic return path forms in the crack. Simultaneously, the magnetic line of force is first leaked into the air and then goes back to the pipe. Subsequently, a magnetic sensor scans the pipe to sense the variation of a magnetic field, which is further analyzed. Finally, the identification and judgment of cracks are achieved based on the RMFT signal.

### 2.2. Finite Element Model

**Figure 3:**
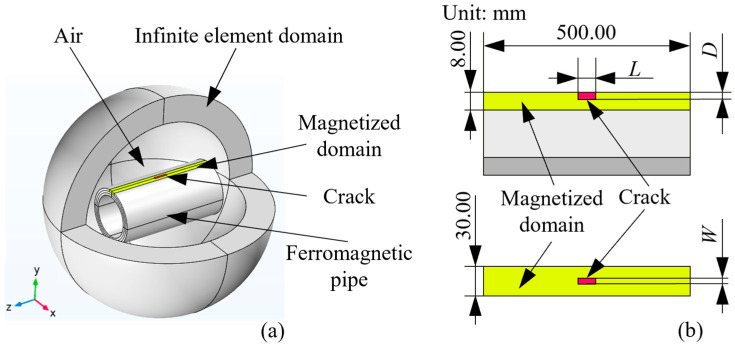
(**a**) Finite element model of RMFT for pipe detection. (**b**) Dimensions and details of the magnetized domain and crack.

To explore the variations in the RMFT signal with the crack size, a finite element model is built by COMSOL 6.0 software, as shown in Figure 3a. The model consists of an air domain, a ferromagnetic pipe with a magnetized domain, and a crack. Furthermore, an infinite element domain (IED) is introduced to simulate the far-field in the model, which can improve solution precision. With respect to the material, the ferromagnetic pipe, including the magnetized domain, is carbon steel, and all remaining components in the model are air. The materials and relative permeabilities of the finite element model are listed in Table 1. The residual magnetic flux density of the magnetized domain is uniformly assumed to be 300.00 Gs, which can simplify the simulation model and reduce the solving time. With respect to dimensions, the diameter, length, and thickness of the pipe are 200.00, 500.00, and 8.00 mm, respectively. The dimension of the crack is varied to investigate its effect on the residual magnetic field. In detail, the length (*L*) is changed from 5.00 to 40.00 mm, with an increment of 5.00 mm. Meanwhile, the depth (*D*) is increased from 1.00 to 8.00 mm, with a step of 1.00 mm. The lateral dimension of the crack, namely, the width (*W*), is fixed at 1.00 mm, and the detailed dimensions and position of the crack are represented in Figure 3b. The dimensions of different domains in the finite element model are summarized in Table 2. The free tetrahedral element is selected to mesh all domains in the finite element model. The maximum element size of the crack domain is 0.10 mm, and the other domains are automatically meshed at a finer level by COMSOL software. When *L* = 30.00 mm and *D* = 5.00 mm, 283,946 elements are generated in the model.

A part of the pipe, labeled in yellow in Figure 3a, is taken out as the magnetized domain, and the detailed dimensions of the magnetized domain are shown in Figure 3b. A residual magnetic flux density along the circumferential direction of the pipe is applied to the magnetized domain to simulate the residual magnetic effect, which is caused by the passage of the permanent magnet. Therefore, the width of the magnetized domain and that of the permanent magnet are consistent. The *x*-directional magnetic signal (*B_x_*) attributed to the crack is obtained through a cut line, which is along the axial direction of the pipe and is also the motion path of the magnetic sensor in the real inspection. The distance between the data line and the pipe surface is 7.00 mm, which is the sum of the lift-off distance (2.00 mm) and the coating thickness (5.00 mm). It should be noted that the coating is not considered in the model. This is because the material of coating (nylon) has the same electromagnetic properties as air.

### 2.3. Signature Waveform Analysis

**Figure 4:**
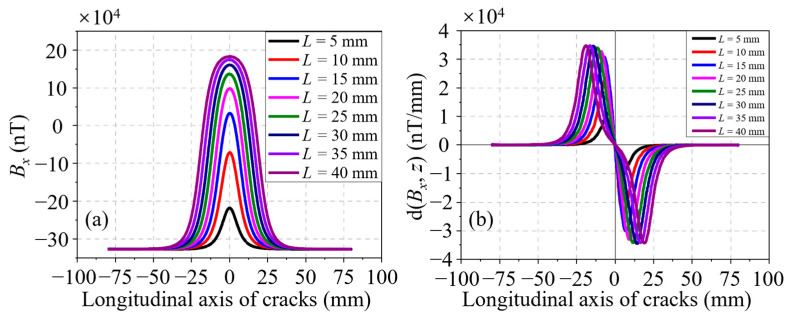
(**a**) *B_x_* curves of cracks at different lengths along the *z*-axis. (**b**) d(*B_x_*, *z*) curves of cracks at different lengths along the *z*-axis.

**Figure 5:**
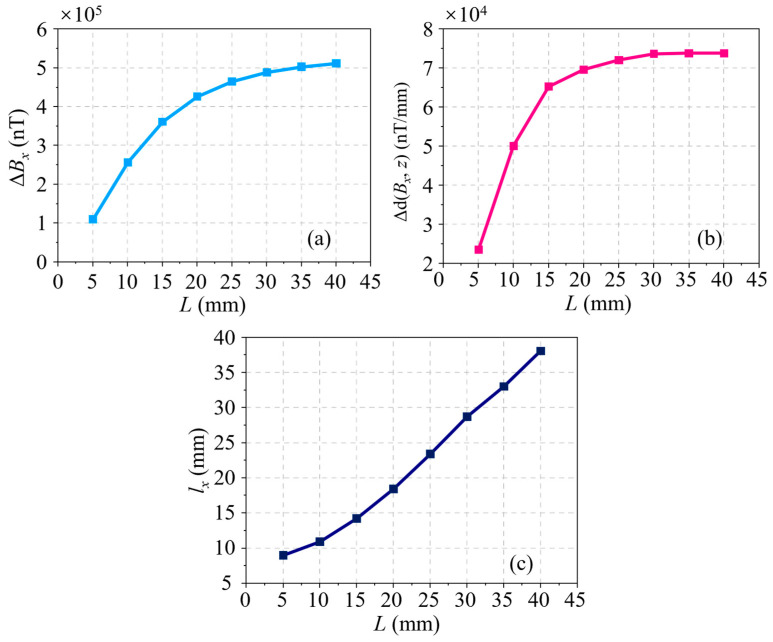
(**a**) Δ*B_x_* variation with *L* based on simulations. (**b**) Δd(*B_x_*, *z*) variation with *L* based on simulations. (**c**) *l_x_* variation with *L* based on simulations.

Through analyzing the *B_x_* signals for a series of cracks, which have different lengths but a fixed depth of 4.00 mm, the effect of the crack length on the residual magnetic signal is first acquired. A set of *B_x_* signals at different lengths of *L* ranging from 5.00 to 40.00 mm, with an increment of 5.00 mm, is shown in Figure 4a. It is evident that the *B_x_* curve is sensitive to the variation of *L*. At length, both the amplitude and the waveform width of the *B_x_* curve increase with *L*. Partial derivatives are taken with respect to the axial direction of the pipe (*z*-axis) to obtain the d(*B_x_*, *z*) curves, as illustrated in Figure 4b. It can be seen that the waveform width also increases with *L*, whereas the discrimination in amplitude between different curves is fuzzy. The characteristics in the residual magnetic signal, namely, the peak-to-peak value in the *B_x_* curve Δ*B_x_*, the peak-to-peak value in the d(*B_x_*, *z*) curve Δd(*B_x_*, *z*), and the distance between the peak and trough in the d(*B_x_*, *z*) curve *l_x_*, are extracted. Moreover, the relationships between these characteristics and *L* are displayed in Figure 5a–c, respectively. As shown in Figure 5a,b, the characteristics Δ*B_x_* and Δd(*B_x_*, *z*) have a similar trend, that is, they increase quickly at first and then slowly with the crack length, and their growth rates descend gradually. Unlike the two characteristics analyzed in advance, Figure 5c shows that the correlation between *l_x_* and *L* is approximately linear, which is a tremendous advantage for the representation of the crack size.

**Figure 6:**
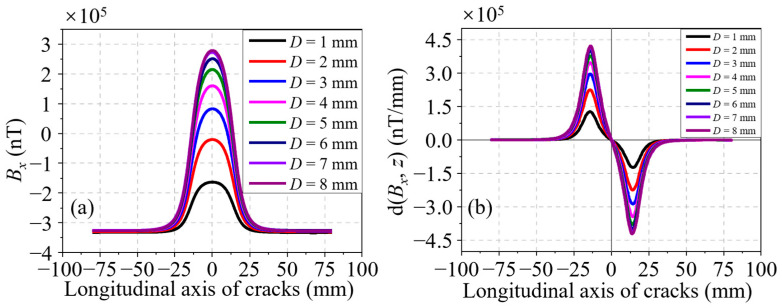
(**a**) *B_x_* curves of cracks at different depths along the *z*-axis. (**b**) d(*B_x_*, *z*) curves of cracks at different depths along the *z*-axis.

**Figure 7:**
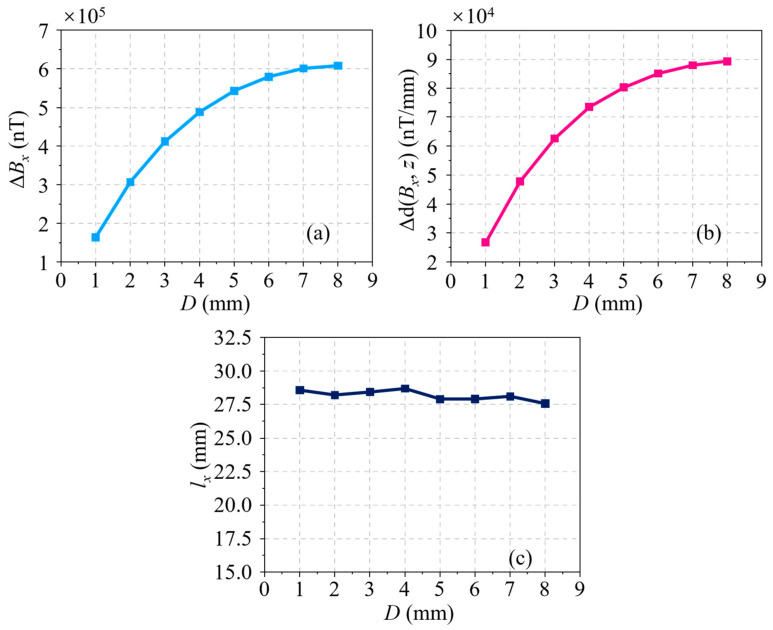
(**a**) Δ*B_x_* variation with *D* based on simulations. (**b**) Δd(*B_x_*, *z*) variation with *D* based on simulations. (**c**) *l_x_* variation with *D* based on simulations.

Next, the influence of the crack depth on the residual magnetic signal is then analyzed at a constant length of 30.00 mm and different depth, which increases from 1.00 to 8.00 mm at a step of 1.00 mm. The *B_x_* curves and their corresponding d(*B_x_*, *z*) curves of all cracks are shown in Figure 6a,b, respectively. In amplitude, it is not hard to find that the two curves rise with *D*. In waveform width, it is not easy to identify their differences in both the *B_x_* and d(*B_x_*, *z*) curves. To better understand the variation of the residual magnetic signal with *D*, the characteristics Δ*B_x_* in the *B_x_* curves and two characteristics Δd(*B_x_*, *z*) and *l_x_* in the d(*B_x_*, *z*) curves are taken out. The variation curves of all characteristics with *D* are revealed in Figure 7a–c. Obviously, a monotonic increasing trend can be found in the variation curves of characteristics Δ*B_x_* and Δd(*B_x_*, *z*), as shown in Figure 7a,b. It can be seen from Figure 7c that the *l_x_* variation curve is a nearly horizontal line. In other words, the characteristic *l_x_* is independent of *D*. Accordingly, *l_x_* cannot be used to represent the crack depth.

According to the simulation results, all three characteristics are able to respond to the change in the crack length, and *l_x_* is more sensitive than the other two characteristics (Δ*B_x_* and Δd(*B_x_*, *z*)) for the variation of *L*. The characteristics Δ*B_x_* and Δd(*B_x_*, *z*) are monotonically related to the crack depth, which cannot play a role in the characteristic *l_x_*. In summary, it is demonstrated that the RMFT method can be used to inspect cracks in ferromagnetic pipes from the perspective of the simulation.

## 3. Experimental Setup and Results

### 3.1. RMFT System

**Figure 8:**
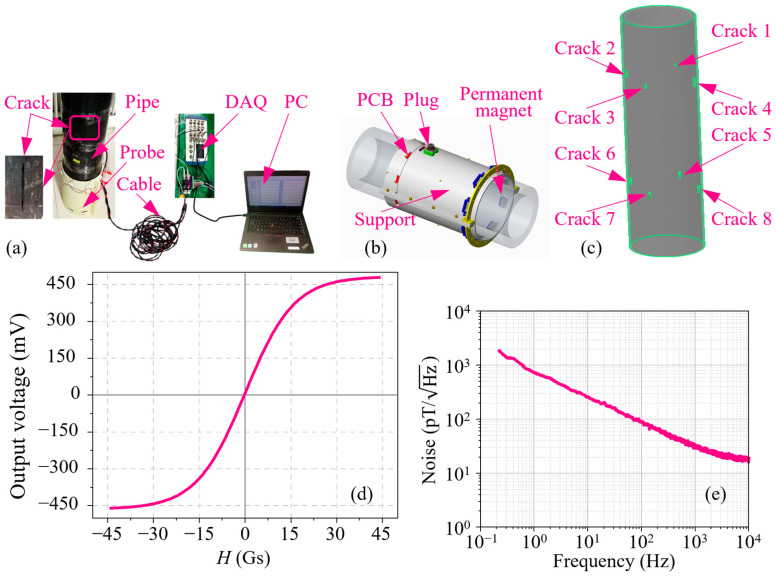
(**a**) Setup of RMFT experimental system. (**b**) RMFT probe. (**c**) Cracks machined in carbon steel pipe. (**d**) Sensitivity curve of TMR sensor. (**e**) Noise power spectral density of TMR sensor.

A photograph of the RMFT system is shown in Figure 8a; there is a detection probe, a data transmission cable, a data acquisition card (DAQ), and a PC in the system. These modules play different roles in detecting defects in pipes. In testing, a carbon steel pipe (20 steel, which meets Chinese standard GB/T 8163-2018 [23]) is scanned by the probe, which is used to magnetize the pipe and collect the analog signal, synchronously. The cable transmits the signal to the DAQ (NI 6218-BNC, National Instrument, Austin, TX, USA), which is used to disperse the analog signal to the digital signal with a sampling frequency of 100 Hz. The raw signal is further denoised by a PC using some algorithms, such as the empirical mode decomposition (EMD) [24] method.

In the RMFT system, the probe is a key equipment, which not only provides the magnetized source for the pipe but also acquires the residual magnetic signal. The probe consists of 8 permanent magnets, 8 TMR sensors, a plug, a support, and some adjusting screws, as shown in Figure 8b. Figure 8d displays the sensitivity curve of the TMR sensor (MultiDimension Technology, Suzhou, China). It can be seen that the sensitivity of the TMR sensor is 30 mV/V/Gs, which is determined by the slope in the transferring curve with a linear range of *H* = ±10 Gs. Another significant parameter of the TMR sensor is intrinsic noise, which determines the detection precision of the probe. As can be shown in Figure 8e, the noise of the TMR sensor decreases with frequency gradually. The self-noise of the sensor is 750 $pT/\sqrt{Hz}$ at *f* = 1 Hz, which is a relatively low level compared with coils and other magnetic sensors. A TMR sensor is attached to a printed circuit board (PCB), and the corresponding voltage amplifier circuit is designed using a gain transferring function of 100 times over a low-pass filter at a frequency *f* ≤ 20 Hz. A sensing array including 8 signal acquisition circuits is developed. The array is placed at the top of the support to improve detection efficiency. In addition, the sensitive direction of every TMR sensor is tangent to the pipe. There are 8 permanent magnets with a residual magnetic flux density of 1.20 kGs in the exciting module, which is installed at the end of the support. The magnetic flux density in the permanent magnet is also along the circumferential direction of the pipe. The centers of a TMR sensor and the corresponding permanent magnet can be connected as a straight line, which is parallel to the axial line of the pipe. The distance between the TMR sensor and the corresponding magnet is 310.00 mm, which is large enough to eliminate the interference with each other. Furthermore, the plug is applied to connect the PCBs and the cable. The adjusting screws have the function to hold the distance between the sensors and the pipe, which is also the lift-off distance. In experiments, the lift-off is set as 2.00 mm. In testing, the residual magnetic field is retained in the pipe due to the scanning of the magnet. Hence, the pipe should be demagnetized to eliminate the effect of the residual magnetic field in advance.

To verify the developed RMFT system, a series of cracks with a width of 1.00 mm are machined by a drill press at the surface of the new carbon steel pipe, and these cracks are labeled No. 1 to 8, respectively. The positions of all cracks are shown in Figure 8c, and their dimensions are summarized in Table 3. It is worth noting that the depths of all the cracks are the values in the metal layer of the pipe. From the pipe surface, the depths should be uniformly added 50.00 mm, which is the thickness of the coating. The other parameters of the pipe are the same as the simulation model.

### 3.2. Results Analysis and Discussion

**Figure 9:**
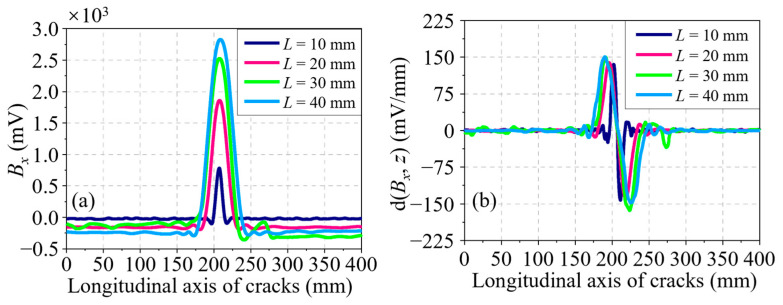
(**a**) *B_x_* signals of cracks 1–5. (**b**) d(*B_x_*, *z*) signals of cracks 1–5.

**Figure 10:**
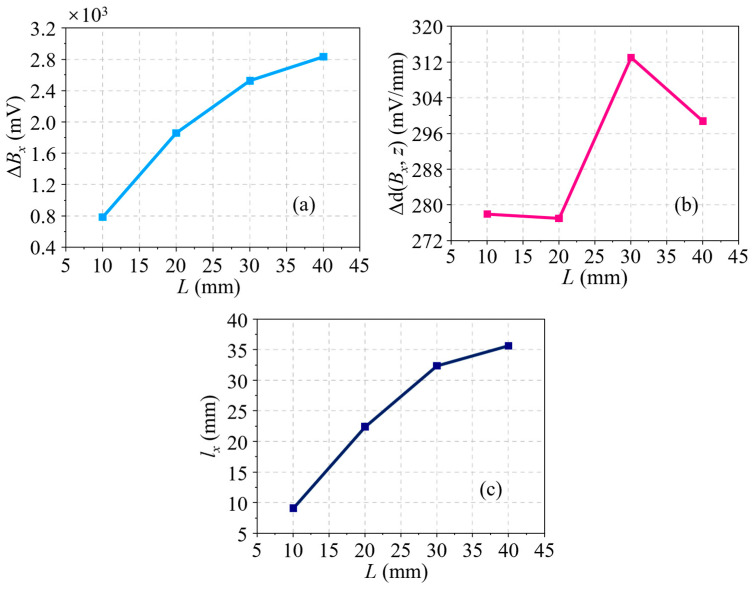
(**a**) Δ*B_x_* variation with *L* based on experiments. (**b**) Δd(*B_x_*, *z*) variation with *L* based on experiments. (**c**) *l_x_* variation with *L* based on experiments.

The effect of the crack length is first analyzed through inspecting the first group cracks (No. 1–5). During measurement, the probe is moved along the axial direction of the pipe, i.e., the longitudinal direction of all cracks. The *B_x_* signal of each channel in the sensor array is extracted and shown in Figure 9a. It can be seen from the figure that the distinction is obvious in these *B_x_* curves of the cracks with different lengths. Similar to the simulations, all *B_x_* signals are taken derivatives with respect to the *z*-axis, and the corresponding d(*B_x_*, *z*) curves are shown in Figure 9b. Unlike the *B_x_* signals, it is difficult to directly distinguish these d(*B_x_*, *z*) curves. Then, the characteristics Δ*B_x_*, Δd(*B_x_*, *z*), and *l_x_* are retrieved, and their corresponding variation curves with the crack length are illustrated in Figure 10a–c, respectively. As shown in Figure 10a, the Δ*B_x_* values of cracks 1–4 are 0.79 × 10^3^, 1.86 × 10^3^, 2.53 × 10^3^, and 2.83 × 10^3^ mV, respectively. It is known that the distortion value in the *B_x_* signal increases as the crack length increases, which coincides with the simulation results. Figure 10b indicates that the Δd(*B_x_*, *z*) variation is irregular with *L*. The reasons may be the uneven surface in the pipe and the probe vibration. Based on the experimental results, the crack size is hard to predict using the characteristic Δd(*B_x_*, *z*). The characteristics *l_x_* are determined to be 9.12, 22.44, 32.40, and 35.70 mm for cracks 1–4, respectively. It is inferred that the *l_x_* value increases with the increase of *L*, but the growth rate gradually becomes smooth, which is a slight difference from the simulations. Anyway, the characteristic *l_x_* can be used to express the crack length.

**Figure 11:**
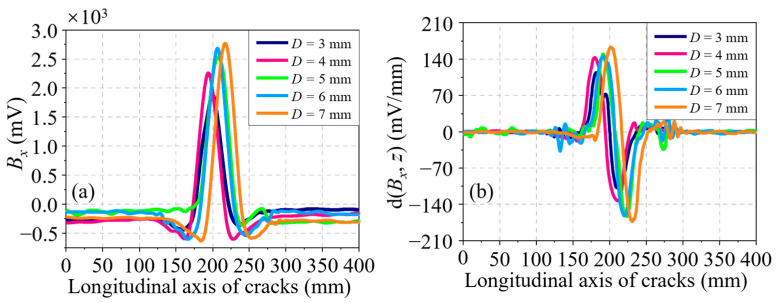
(**a**) *B_x_* signals of cracks 2 and 5–8. (**b**) d(*B_x_*, *z*) signals of cracks 2 and 5–8.

**Figure 12:**
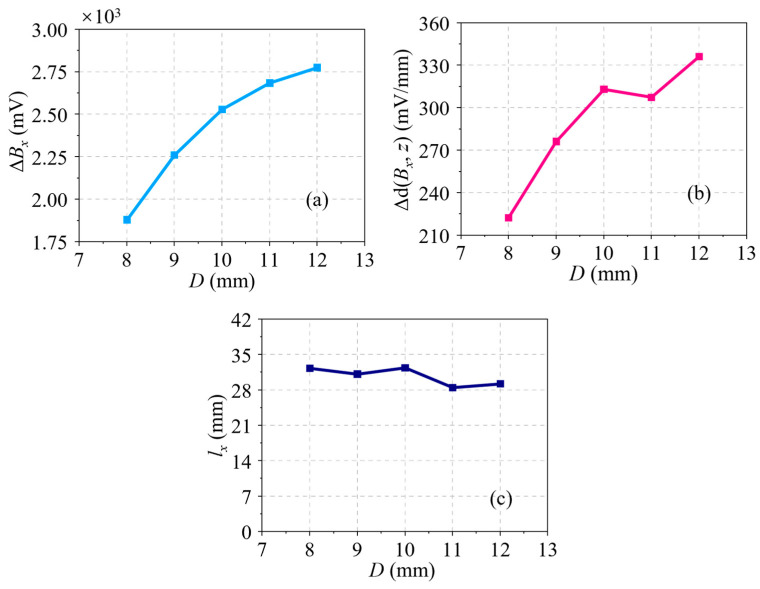
(**a**) Δ*B_x_* variation with *D* based on experiments. (**b**) Δd(*B_x_*, *z*) variation with *D* based on experiments. (**c**) *l_x_* variation with *D* based on experiments.

Subsequently, the influence of the crack depth is investigated using cracks 2 and 5–8, and their corresponding *B_x_* signals are presented in Figure 11a, where these *B_x_* curves can be clearly distinguished. Particularly, the peak-to-peak values in all *B_x_* curves are measured to be 1.88 × 10^3^, 2.26 × 10^3^, 2.53 × 10^3^, 2.69 × 10^3^, and 2.77 × 10^3^ mV, respectively. Meanwhile, the relationship between Δ*B_x_* and *D* is depicted in Figure 12a. It is observed that the Δ*B_x_* value become larger as *D* increases, but the increment declines step by step, which agrees with the simulation results. Conventionally, the differentials of all *B_x_* signals about the *z*-axis are calculated, and the resulting curves are shown in Figure 11b. Obviously, they are distinct enough to be identified, but it is not easy to find the differences of characteristics *l_x_* in these d(*B_x_*, *z*) curves. Hence, the characteristics Δd(*B_x_*, *z*) and *l_x_* of all d(*B_x_*, *z*) curves in Figure 11b are fetched out and their changes with *D* are displayed in Figure 12b,c. As shown in Figure 12b, in contrast to the simulation results, the Δd(*B_x_*, *z*) curve is tortuous rather than monotonous. In other words, the characteristic Δd(*B_x_*, *z*) can not signify the crack depth based on the experimental results. Figure 12c shows that the characteristic *l_x_* is insensitive to the crack depth, and the *l_x_* values of all cracks are 32.30, 31.16, 32.40, 28.48, and 29.22 mm, respectively. The experimental results of the *l_x_* variation with *D* are consistent with the simulations.

In accordance with the experiments, the changing trend of the characteristic Δ*B_x_* with both the crack length and depth is monotonically increasing. Nevertheless, the variation of the characteristic Δd(*B_x_*, *z*) is uncertain not only with the crack length but also with the depth. Meanwhile, the characteristic *l_x_* depends on the crack length positively but is independent of the crack depth. To sum up, on the basis of the relationship between the characteristics in the residual magnetic signals (*l_x_* and Δ*B_x_*) and the crack size (*L* and *D*), the profile of the crack can be easily portrayed using the polynomial fitting [25] or the machine learning algorithm [26], which have already been applied in ACFM. In short, the feasibility of crack detection using the RMFT method is verified by the experiments.

## 4. Conclusions

In this paper, the RMFT probe with TMR sensor arrays and the testing system for crack detection in ferromagnetic pipes are proposed. A finite element model is established, and the perturbed residual magnetic signals are simulated. The simulation results demonstrate that both residual magnetic signal (*B_x_*) and its differential with respect to the axial direction of the pipe (d(*B_x_*, *z*)) can be influenced by the crack dimensions (length and depth) significantly. Moreover, the crack profile can be depicted by three characteristics (Δ*B_x_*, Δd(*B_x_*, *z*), and *l_x_*). Based on the simulation results, a sensing element is developed based on the TMR sensor, which has a high sensitivity of 30.00 mV/V/Gs in a range of ±10 Gs and a noise of 750 $pT/\sqrt{Hz}$ at 1 Hz. The PCBs are printed on the condition of a gain-transferring function of 100 times and a low-pass filter of 20 Hz. Such high-sensitive and low-noised TMR sensors are assembled into an array probe, which is found to be adaptive for weak residual magnetic field detection. An experiment is carried out, and the residual magnetic signals of cracks are analyzed. The results demonstrate that the variations of the characteristics Δ*B_x_* and *l_x_* with the crack size are in agreement with the simulations, which also verifies that the RMFT is a feasible method for crack detection in ferromagnetic pipes.

The proposed RMFT method provides a low-cost, compact-size, and low-power testing solution, which is the first step toward the optimization of the RMFT system. In practice, it should be possible to detect the oil and gas pipeline using a novel RMFT probe, which is integrated into a pipe pig to inspect the inner surface of the pipe. At present, we are designing a novel probe to inspect cracks in the inner surface of the pipe and developing a quantification algorithm for crack sizing based on RMFT technology, which will be presented in our future work.

## Figures and Tables

**Table 1:** Materials and electromagnetic parameters of finite element model.

Model	Material	Relative Permeability	Residual Magnetic Flux Density (Gs)
Infinite element domain	Air	1	-
Magnetized domain	Carbon steel	1000 [22]	300.00
Pipe	Carbon steel	1000	-
Crack	Air	1	-
Others	Air	1	-

**Table 2:** Dimensions of finite element model.

Model	Diameter (mm)	*L* (mm)	*W* (mm)	*D* (mm)	Thickness (mm)
Infinite element domain	1000.00	-	-	-	100.00
Magnetized domain	-	500.00	30.00	8.00	-
Pipe	200.00	500.00	-	-	8.00
Crack	-	Varied	1.00	Varied	-

**Table 3:** Dimensions of cracks in the pipe.

Crack	*L* (mm)	*D* (mm)	Crack	*L* (mm)	*D* (mm)
1	10.00	5.00	5	30.00	7.00
2	30.00	5.00	6	30.00	6.00
3	20.00	5.00	7	30.00	4.00
4	40.00	5.00	8	30.00	3.00

## Data Availability

Data are contained within the article. The data that support the findings of this study are available from the corresponding author, upon reasonable request.

## References

[1] Sharma S.K., Maheshwari S. (2017). A review on welding of high strength oil and gas pipeline steels. J. Nat. Gas Sci. Eng..

[2] Kim M., Ha J., Kim Y.-T., Choi J. (2022). Stainless steel: A high potential material for green electrochemical energy storage and conversion. Chem. Eng. J..

[3] Shi Y., Zhang C., Li R., Cai M., Jia G. (2015). Theory and Application of Magnetic Flux Leakage Pipeline Detection. Sensors.

[4] Piao G., Guo J., Hu T., Leung H., Deng Y. (2019). Fast reconstruction of 3-D defect profile from MFL signals using key physics-based parameters and SVM. NDT E Int..

[5] Wang Z.D., Gu Y., Wang Y.S. (2012). A review of three magnetic NDT technologies. J. Magn. Magn. Mater..

[6] Zhao S., Shen Y., Wang J., Jiang Z., Mao Z., Chu Z., Gao J. (2023). A Defect Visualization Method Based on ACFM Signals Obtained by a Uniaxial TMR Sensor. IEEE Sens. J..

[7] Wu J., Yang F., Jing L., Liu Z., Lin Y., Ma H. (2022). Defect detection in pipes using Van der Pol systems based on ultrasonic guided wave. Int. J. Press. Vessel. Pip..

[8] Feng Q.S., Li R., Nie B.H., Liu S.C., Zhao L.Y., Zhang H. (2017). Literature Review: Theory and Application of In-Line Inspection Technologies for Oil and Gas Pipeline Girth Weld Defection. Sensors.

[9] Yu Z., Fu Y., Jiang L., Yang F. (2021). Detection of circumferential cracks in heat exchanger tubes using pulsed eddy current testing. NDT E Int..

[10] Zhao J., Li W., Yuan X., Yin X., Ding J., Chen Q., Yang H. (2023). Rotating alternating current field measurement testing system with TMR arrays for arbitrary-angle crack on nonferromagnetic pipes. Meas. Sci. Technol..

[11] Hayt W.H., Buck J.A. (2012). Engineering Electromagnetics.

[12] Betta G., Ferrigno L., Laracca M., Rasile A., Sangiovanni S. (2021). A novel TMR based triaxial eddy current test probe for any orientation crack detection. Measurement.

[13] Zhao S., Shen Y., Sun L., Wang J., Mao Z., Chu Z., Chen J., Gao J. (2022). A method to compensate for the lift off effect of ACFM in crack estimation of nonferromagnetic metals. J. Magn. Magn. Mater..

[14] Lee J., Jun J., Kim J., Choi H., Le M. (2012). Bobbin-Type Solid-State Hall Sensor Array With High Spatial Resolution for Cracks Inspection in Small-Bore Piping Systems. IEEE Trans. Magn..

[15] He D.F., Shiwa M., Jia J.P., Takatsubo J., Moriya S. (2011). Multi-frequency ECT with AMR sensor. NDT E Int..

[16] Ye C.F., Huang Y., Udpa L., Udpa S.S. (2016). Novel Rotating Current Probe With GMR Array Sensors for Steam Generate Tube Inspection. IEEE Sens. J..

[17] Yuan X., Li W., Chen G., Yin X., Jiang W., Zhao J., Ge J. (2019). Inspection of both inner and outer cracks in aluminum tubes using double frequency circumferential current field testing method. Mech. Syst. Signal Process..

[18] Hayakawaa J., Ikeda S., Lee Y.M., Matsukura F., Ohno H. (2006). Effect of high annealing temperature on giant tunnel magnetoresistance ratio of CoFeB/MgO/CoFeB magnetic tunnel junctions. Appl. Phys. Lett..

[19] Li X.Y., Hu J.P., Chen W.P., Yin L., Liu X.W. (2018). A Novel High-Precision Digital Tunneling Magnetic Resistance-Type Sensor for the Nanosatellites’ Space Application. Micromachines.

[20] Yang L., Cui W., Liu B., Gao S. (2015). A pipeline defect detection technique based on residual magnetism effect. Oil Gas Storage Transp..

[21] Chen D.-X., Pardo E., Zhu Y.-H., Xiang L.-X., Ding J.-Q. (2018). Demagnetizing correction in fluxmetric measurements of magnetization curves and hysteresis loops of ferromagnetic cylinders. J. Magn. Magn. Mater..

[22] Ge J., Li W., Chen G., Yin X., Wu Y., Liu J., Yuan X. (2017). Analysis of signals for inclined crack detection through alternating current field measurement with a U-shaped probe. Insight-Non Destr. Test. Cond. Monit..

[23] (2018). Seamless Steel Pipes for Liquid Service.

[24] Chen L., Li X.B., Qin G.X., Lu Q. (2008). Signal processing of magnetic flux leakage surface flaw inspect in pipeline steel. Russ. J. Nondestruct. Test..

[25] Zhao S., Sun L., Gao J., Wang J., Shen Y. (2020). Uniaxial ACFM detection system for metal crack size estimation using magnetic signature waveform analysis. Measurement.

[26] Zhao S., Shen Y., Wang J., Zhu R., Zhai W., Dong H., Mao Z., Gao J. (2022). Extreme learning machine based sub-surface crack detection and quantification method for ACFM. J. Magn. Magn. Mater..

